# Synthesis of thermo-responsive polymer gels composed of star-shaped block copolymers by copper-catalyzed living radical polymerization and click reaction

**DOI:** 10.1080/14686996.2024.2302795

**Published:** 2024-02-14

**Authors:** Guohao Gao, Mitsuo Hara, Takahiro Seki, Yukikazu Takeoka

**Affiliations:** Department of Molecular & Macromolecular Chemistry, Nagoya University, Nagoya, Japan

**Keywords:** Thermo-responsive polymer, star-shaped polymer, polymer networks, gels, end-group modification

## Abstract

In recent times, there has been a significant surge in research interest surrounding thermo-responsive water-soluble polyacrylamides, primarily due to their intriguing capability to undergo significant solubility changes in water. These polymers exhibit the remarkable ability to shift from a soluble to an insoluble state in response to temperature variations. The capacity of these polymers to dynamically respond to temperature changes opens up exciting avenues for designing smart materials with tunable properties, amplifying their utility across a spectrum of scientific and technological applications. Researchers have been particularly captivated by the potential applications of thermo-responsive water-soluble polyacrylamides in diverse fields such as drug delivery, gene carriers, tissue engineering, sensors, catalysis, and chromatography separation. This study reports the construction and functionalization of polymer gels consisting of a polymer network of polyacrylamide derivatives with nano-sized structural units. Specifically, thermo-responsive polymer gels were synthesized by combining well-defined star-shaped polymers composed of polyacrylamide derivatives with a multifunctional initiator and linking method through a self-accelerating click reaction. The polymerization system employed a highly living approach, resulting in polymer chains characterized by narrow molecular weight distributions. The method’s high functionality facilitated the synthesis of a temperature-responsive block copolymer gel composed of *N*-isopropyl acrylamide (NIPA) and *N*-ethyl acrylamide (NEAA). The resulting polymer gel, comprising star-shaped block copolymers of NIPA and NEAA, showcases smooth volume changes with temperature jumps.

## IMPACT STATEMENT

This approach’s versatility was showcased by creating networks using widely-used vinyl polymers. It can generate various functional and nearly ideal gels and elastomers, allowing for investigating fundamental aspects of polymer networks.

## Graphical abstract

**Figure:**
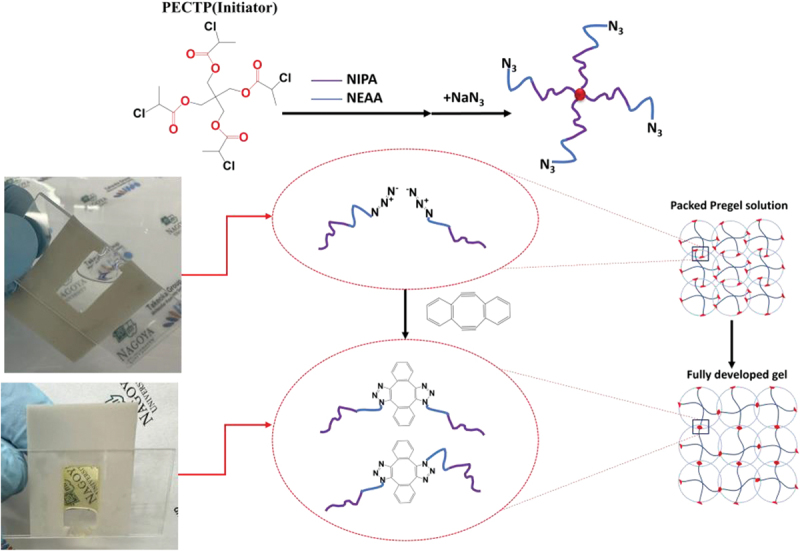

## 1. Introduction

The stimulus-responsive polymer gel represents a versatile and functional soft material that undergoes reversible volume changes in response to environmental variations, including temperature, pH, light exposure, and electric field application [1–3]. Beyond altering its volume, this type of polymer gel exhibits reversible shifts in solvent retention capacity, solute diffusivity, molecular recognition ability, catalytic reactivity, and surface substance adsorption. By leveraging these distinctive properties, researchers have enthusiastically explored the applications of stimulus-responsive polymer gels in fields such as actuators [4], artificial enzymes [5], and sensors [6,7].

As a material for preparing such stimulus-responsive gels, polyacrylamide derivatives are attractive polymers that undergo rapid changes in solubility in aqueous solution. Specifically, depending on temperature, these polymers can switch from a soluble state at low temperatures to an insoluble state at high temperatures in aqueous solution [8–10], exhibiting the so-called lower critical solution temperature (LCST). Poly(*N*-isopropyl acrylamide) (PNIPA) is one of the most studied polyacrylamide derivatives because it shows a pronounced LCST in water at around 32°C. At low temperatures, PNIPA is hydrophobically hydrated at the main chain and at the hydrophobic portion of the *N*-substituent and hydrated by hydrogen bonding at the amide group, making it soluble in water. Water molecules in a hydrophobically hydrated state exhibit negative entropy when PNIPA mixes with water because they have reduced entropy compared to bulk water molecules. At low temperatures, such as room temperature, the negative enthalpy term due to the hydration effect of PNIPA dominates the Gibbs free energy, causing PNIPA to dissolve in water. However, at temperatures higher than LCST, the entropy term becomes dominant, and PNIPA releases water, causing phase separation. The phase separation temperature of the associated polyacrylamide can be significantly altered by changing the hydrophobicity of the *N*-substituents on the monomer unit.

Recent advances in polymer synthesis methods that allow easy control of precise structure have enabled the precise synthesis of thermo-responsive polymers and their polymer gels [11–17]. For example, polymer synthesis by radical polymerization mediated by copper catalysts, a tooled polymerization reaction, combined with polymer chain modification techniques using click reactions, allows the construction of polymers of various shapes and polymer networks [18–21]. As a result, they have been used to produce hyperbranched and highly branched polymers and bottlebrush polymers with controlled molecular weight and polymer networks composed of these polymers. However, in using these reactions, appropriate reaction conditions must be selected.

Copper-mediated living polymerization enables the acquisition of polymers with controlled chain length and narrow molecular weight distributions, provided appropriate reaction conditions – such as initiator, solvent, and temperature – are meticulously chosen for the targeted monomer [22–24]. However, achieving precise control, particularly in synthesizing block copolymers, becomes challenging when polymerizing diverse monomers under identical conditions. Attaining high functionality by converting polymer chain end groups, initiated with alkyl halide groups, necessitates the stability of halogenated chain ends throughout polymerization. Notably, when crafting block copolymers with acrylamide derivatives, a discernible increase in the disappearance of halogenated end groups is observed for tertiary acrylamide compared to secondary acrylamide. This divergence is ascribed to the electron density of the amide group, which intensifies with substantial alkyl substitution on the nitrogen atom. The elevated electron density, influenced by inductive effects, promotes cyclization reactions of halogenated end groups, leading to faster termination and the loss of active polymer chains. Unfortunately, copper complexes employed in polymerization face challenges in effectively activating these species, impeding conversion and substitution reactions of halogenated end groups. This complexity compromises polymerization control and impedes the desired end-group modification. Consequently, it is prudent to opt for secondary acrylamide derivatives when synthesizing block copolymers. If a reactive alkyl halide remains at the polymer chain’s end, subsequent modifications can introduce new structures or functions to the polymer.

The click reaction is very effective for modifying the ends of polymer chains. One of the outstanding contributions in the click reaction is the introduction of a double strain-promoted azide – alkyne cycloaddition (DSPAAC) reaction through the bimolecular homodifunctional method, utilizing sym-dibenzo-1,5-cyclooctadiene-3,7-diyne (DIBOD) as the small difunctional linker [25,26]. DSPAAC’s distinct advantage lies in its self-accelerating nature, surpassing traditional azide – alkyne click reactions. DIBOD activates the second alkynyl group in situ in this reaction, resulting in an accelerated second-step reaction with a much higher rate than the first step. This self-accelerating DSPAAC reaction is suitable for polymer chain dimerization, breaking through equimolar feeding restrictions. Excess DIBOD significantly increases the intermolecular coupling reaction rate and enhances dimerization efficiency. Furthermore, unlike the widely used copper complex reaction, DSPAAC can be efficiently conducted under mild conditions, such as in the air or at room temperature, without needing catalysts or chemical stimuli.

This study presents a simple and tunable method to produce star-polymer-based gels from two acrylamide derivative polymers. Our approach involves only two steps: The synthesis of star-shaped polymers consisting of end-active block copolymers in one pot by controlled polymerization and end-group azidation and end-linking synthesis of the resulting star-shaped polymers by a click reaction without using copper complexes – first, copper-meditated living polymerization synthesized monodisperse star-shaped vinyl polymers from multifunctional chlorine initiators. As for monomers, NIPA and *N*-ethyl acrylamide (NEAA) were chosen as secondary acrylamide derivative monomers that provide water-soluble polymers. After the polymerization reaction was sufficiently advanced, sodium azide was added in the same vessel to directly convert the alkyl chloride end group of the resulting star polymer to an azide group. Next, star-shaped polymers with azide ends were prepared under solution conditions that allowed efficient linking of the ends between different star-shaped polymers, and then cyclooctadiyne linkers were reacted with the ends of the star-shaped polymers to form gels using strain-promoted azide-alkyne click chemistry. The resulting diblock copolymer of the above two monomers dimerized efficiently, yielding a thermo-responsive polymer gel. The polymer network structure obtained by this method is considered more well-organized than that obtained by conventional methods.

## 2. Materials and methods

### 2.1. Materials

Acetone, hexane, tetrahydrofuran (THF), methanol, chloroform, ethyl acetate (EtOAc), ethanol, acetonitrile, acryloyl chloride, sodium hydroxide, potassium hydroxide, *N*, *N*-dimethylformamide (DMF), anhydrous magnesium sulfate, and diethyl ether were purchased from Kanto Chemical. Copper (I) chloride (CuCl), lithium bromide (LiBr), ethylamine, and DIBOD were purchased from the Tokyo Chemical Industry. Sodium azide and dibenzocyclooctyne-amine (DBCO-amine) were purchased from Sigma-Aldrich. *N*, *N*-dimethylformamide (DMF) for preparing the GPC/SEC’s eluent and hydrochloric acid (12 N) were purchased from FUJIFILM Wako Pure Chemical Industry. Pentaerythritol tetra (2-chloropropionate) (PETCP) was commercially obtained as a custom-made product from the Tokyo Chemical Industry and used as an initiator. Ultrapure water (specific resistance value: 18.2 MΩ cm) obtained by purifying tap water with a Direct-Q 3UV manufactured by Millipore was used. In addition, *N*-isopropyl acrylamide (NIPA) and tris (2-dimethylamino ethyl) amine (Me_6_TREN) were kindly provided by Kohjin and Mitsubishi Chemical, respectively. NIPA was purified by recrystallization using a mixture of hexane and acetone. Me_6_TREN (Mitsubishi Chemical) and DMF (Kanto Chemical) for synthesis were purified by vacuum distillation. Furthermore, other chemicals were used without purification.

### 2.2. Purification of CuCl

Using 20 ml of 12 N hydrochloric acid to dissolve 5 g of CuCl in a 1000 ml Erlenmeyer flask, after fully dissolved, diluted with pure water until no more appearance of white sediment and filtered off. Washed with ethanol and diethyl ether three times, then dried in vacuo for 6 hours at 40°C and stored in a vacuum desiccator.

### 2.3. Synthesis of N-ethyl acrylamide (NEAA)

A solution of acryloyl chloride (0.165 mol, 12.74 ml) in 80 ml THF was added dropwise for an hour and a half to the mixture of aqueous ethylamine (0.15 mol, 12.5 ml) in 100 ml THF and sodium hydroxide aqueous solution (0.165 mol in 12 ml water). Furthermore, stirred under −5°C for three hours, then stirred overnight at room temperature. After the reaction, suction filtration proceeded, and then the aqueous phase of the solution was extracted three times with EtOAc. The combined organic phases were dried over MgSO_4_ filtered, and the solvent was removed in vacuo. The crude product was purified by silica column chromatography and stored at 2 ~ 8°C.

### 2.4. Synthesis of the star-shaped block copolymer composed of NIPA and NEAA via copper-mediated living polymerization

A magnetic stir bar, H_2_O (3.8 ml), and Me_6_TREN (193 µL, 0.72 mmol) were charged to a Schlenk tube (No. 1) fitted with a rubber septum, and the solution was deoxygenated via freeze-pump-thaw for six times. The degassed solution was then transferred via cannula to another Schlenk tube (No. 2), previously evacuated and filled with argon, containing CuCl (71.74 mg, 0.72 mmol) and a magnetic stir bar. The mixed suspension became blue immediately after cannula transfer, and massive purple-red color copper (0) powder was found in the suspension. The Schlenk tube (No. 2) was immersed in an ice/water bath, and the reaction mixture was stirred at 0°C for 45 min under argon protection. A magnetic stir bar, H_2_O (5.16 mL), DMF (15.88 ml), PETCP (89.6 mg, 0.18 mmol), and NIPA (3.2590 g, 28.8 mmol) were charged to a Schlenk tube (No. 3) fitted with a rubber septum, and the solution was deoxygenated via freeze-pump-thaw for six times. The final molar ratio of this mixed solution was NIPA: PETCP: Me_6_TREN: Copper (I) chloride (I) = 160: 1: 4: 4. The degassed initiator/monomer solution was then transferred via cannula to the Schlenk tube (No. 2), which had already been immersed in the ice/water bath. The mixed solution was allowed to stir at 0°C for 30 min. A part of the solution of the reaction mixture was then removed for analysis.

The sample for^1^H NMR was directly diluted with acetone-d_6_ (containing 0.03% TMS). In ^1^H NMR, the reaction rate of the monomer at each time was measured. The reaction rate was calculated by taking the integral ratio of the peaks of the vinyl groups of NIPA consumed by the polymerization reaction based on the DMF aldehyde peak (1 H, 8.0 ppm) that does not change with the reaction. The reaction rate was calculated to obtain the integral ratio of the NIPA vinyl group proton peak (1 H, 5.5 ppm). The reaction rate was then calculated from the following formula:

(1) $$\mathrm{Conversion}\left(\%\right)=\left(1-\frac{I_{t}}{I_{0}}\right)\times 100.$$

*I*_t_: Integral ratio of the vinyl group of NIPA to the DMF aldehyde peak at reaction time *t*

*I*_0_: Integral ratio of the vinyl group of NIPA to the DMF aldehyde peak at the preparation time.

The sample solution was diluted with THF, and catalyst residues were removed by filtering through a silica column before DMF SEC analysis. The polymer’s molecular weight and molecular weight distribution obtained via polymerization were measured using a calibration curve using poly (methyl methacrylate) standards.

A magnetic stir bar, H_2_O (2.235 ml), DMF (3 ml), and NEAA (0.765 ml, 7.2 mmol) were charged to a Schlenk tube (No. 4) fitted with a rubber septum, and the solution was deoxygenated via freeze-pump-thaw for six times. The final molar ratio of this mixed solution was NIPA: NEAA: PETCP: Me_6_TREN: CuCl = 160: 40: 1: 4: 4. After the conversion of the polymerization of NIPA was reached above 90%, the degassed monomer solution was then transferred via cannula to the Schlenk tube (No. 2). The mixed solution was allowed to stir at 0°C for 1 hour and 40 min. A part of the solution of the reaction mixture was then removed for analysis. The sample for ^1^H NMR was directly diluted with acetone-d_6_ (containing 0.03% TMS). In ^1^H NMR, the reaction rate of the monomer at each time was measured. The reaction rate was calculated by taking the integral ratio of the peaks of the vinyl groups of NEAA consumed by the polymerization reaction based on the DMF aldehyde peak (1 H, 8.0 ppm) that does not change with the reaction. The reaction rate was calculated to obtain the integral ratio of the vinyl group proton peak (1 H, 5.6 ppm) of NEAA. The sample solution was diluted with THF, and catalyst residues were removed by filtering through a silica column before DMF GPC/SEC analysis. The polymer’s molecular weight and molecular weight distribution obtained via polymerization were measured using a calibration curve using poly (methyl methacrylate) standards.

### 2.5. Synthesis of star-shaped PNIPA-b-PNEAA with azido-ended groups by one-pot reaction

A magnetic stir bar, H_2_O (4 ml), and sodium azide (0.234 g, 7.2 mmol) were charged to a Schlenk tube (No. 5) fitted with a rubber septum, and the solution was deoxygenated via freeze-pump-thaw for six times. Continuing the synthesis of the star-shaped polymer composed of NIPA and NEAA via copper-mediated living polymerization, when the conversion of the polymerization of PNEAA was reached between 90% and 95%, the degassed solution was then transferred via cannula to the Schlenk tube (No. 5). The final molar ratio of this mixed solution was NIPA: NEAA: PETCP: Me_6_TREN: CuCl: NaN_3_ = 160: 40: 1: 4: 4: 40. Then the solution was stirred at room temperature for 24 hours. After the 24-hour reaction, the solution was diluted with THF, and catalyst residues were removed by filtering through a silica column. After that, the resultant sample was dialyzed against methanol for two days a Spectra/Por 6 Diagnosis Membrane MWCO: 3500 (Spectrum Laboratories, Inc.) and against purified water for three to four days. During dialysis, the methanol and purified water were exchanged every day. After dialysis, the sample was freeze-dried with a lyophilizer to obtain a whiter powder. The product was evaluated by GPC/SEC and ^1^H NMR (acetone-d_6_ (containing 0.03% TMS)) measurements.

### 2.6. Synthesis of star-shaped PNIPA-b-PNEAA with DBCO-amine ended group by strain-promoted azide-alkyne cycloaddition (SPAAC) click reaction

DBCO-amine (10 mg, 3.6 × 10^−5^ mol) and 2 ml of acetonitrile were added to a glass tube with a magnetic stir. After DBCO-amine was fully dissolved in acetonitrile, the PNIPA-b-PNEAA ended with azido groups (100 mg, 4.2 × 10^−6^ mol) added. The reaction mixture was stirred at room temperature for 12 hours. After the 12-hour reaction, the resultant sample was dialyzed against methanol for two days a Spectra/Por 6 Diagnosis Membrane MWCO: 3500 (Spectrum Laboratories, Inc.) and against purified water for three to four days. During dialysis, the methanol and purified water were exchanged every day. After dialysis, the sample was freeze-dried with a lyophilizer (FDU- 1200, Tokyo Rika CO., LTD.) for a whiter powder. The product was evaluated by GPC/SEC, FTIR, and ^1^H NMR(acetonitrile-d3) measurements.

### 2.7. Methanolysis degradation of star-shaped PNIPA-b-PNEAA with azido-ended groups

100 mg of star-shaped PNIPA-b-PNEAA with azido-ended groups, 10 mg of KOH, 8 mL of MeOH, and 2 mL of pure water were placed in a 15 mL pressure-resistant test tube and refluxed at 75°C for two days. After the reaction, the solution was neutralized by adding 1 N HCl until the pH showed approximately 7 on the pH test paper. The solution was then dialyzed with ion-exchanged water for three days using dialysis tubes (Spectra/Por6®DialysisbMembrance MWCO: 1,000). After completion of dialysis, the obtained sample was subjected to SEC measurement.

### 2.8. Preparation of PNIPA-b-PNEAA gel by DIBOD

The gel was synthesized using a high concentration (Φ = 1.7 Φ*) of polymer solution in anhydrous acetonitrile, a good solvent for the PNIPA-b-PNEAA chains. Φ* is the concentration at which chain overlap of star-shaped PNIPA-b-PNEAA with azido-ended groups (Φ* ≈ 0.18) occurs, which was estimated using light scattering measurements. 1.1 eq. of DIBOD was dissolved in a small volume (2% volume of solvent) of THF. The polymer and DIBOD solutions were mixed into a clean glass tube, vigorously stirred for 30 seconds, then sealed tightly with parafilm and left for 24 hours. All the preparation procedures were performed at room temperature (approximately 25°C).

### 2.9. Methanolysis degradation of PNIPA-b-PNEAA gel

As described above, PETCP has four ester groups, and the resulting star-shaped polymer can be decomposed into four arm polymers. The obtained gels were decomposed with methanol, and SEC measurement was performed. The experimental operation in the methanol decomposition is the same as that of the star-shaped PNIPA-b-PNEAA with the azido-ended group’s system, so it is omitted.

### 2.10. Characterization techniques

#### 2.10.1. Proton nuclear magnetic resonance spectroscopy

JNM-ECS400-A(JEOL) was used for the ^1^H NMR measurement. Acetone-d_6_ (containing 0.05 vol% TMS) from FUJIFILM Wako Pure Chemical Industries Co., chloroform-d (containing 0.03% TMS) from Kanto Chemical Co., and acetonitrile-d_3_ from Tokyo Chemical Industry Co., were used for the ^1^H NMR measurement.

### 2.11. Size exclusion chromatography

As measuring instruments, the LC-20AD, SIL-20AHT, RID-10A, and CTO-20A from Shimadzu were used as a pump, an autosampler, a detector, and a thermostat, respectively. PROTEIN KW-804 manufactured by Shodex was used by connecting five in series. The eluent was five mM LiBr dissolving in DMF. The flow rate of the eluent at the time of measurement was 1.0 ml/min, and the oven temperature was 40°C. The poly (methyl methacrylate) standards (PMMA, *M*_n_ = 2k, 4k, 8k, 10k, 20k, 50k, 100k, 150k, 2480k) were purchased from Sigma-Aldrich. In measuring the target polymers, the samples were dissolved in the DMF/LiBr mixed solvent, and the solution’s concentration would be 2 wt%.

### 2.12. Fourier transform infrared spectroscopy

The Fourier transform infrared spectrophotometer Nicolet iS50 (Thermo Scientific) was used to measure IR spectra. The number of scans was 32, and the data interval was 0.482 cm^−1^. The OMNIC software was used for the analysis. The measurement method used was the KBr method. The pellets for measurement were sufficiently obtained from crushed 10 mg of the sample with 150 mg of KBr in an agate bowl, placed in a tablet shaper, and pressed into tablets using a small hydraulic press (Specac).

### 2.13. Dynamic light scattering (DLS) measurements

DLS measurements were performed using Malvern’s Zetasizer Nano ZS (He-Ne laser, beam wavelength 633 nm) or the ALV 5000 static and dynamic light scattering system (ALV) (He-Ne laser, beam wavelength 632.8 nm). Twelve measurements with the Zetasizer Nano ZS were made at a scattering angle of 173 ° for 10 s at 20°C. Measurements with the ALV 5000 were performed 720 or 7200 times at a scattering angle of 90 ° for 60 seconds at room temperature.

### 2.14. Dynamic mechanical analysis

The dynamic mechanical analysis of each gelation process was conducted using a rheometer (RSA-G2, TA Instrument). The shear strain amplitude, frequency, and temperature were 2%, 1 Hz, and 25°C, respectively.

### 2.15. Temperature dependence of gel equilibrium swelling

A smartphone was used to take a picture to observe the degree of swelling of the gel. In order to keep the temperature of the gel constant, water in a constant temperature state flowed through a two-layer cell containing a cylindrical gel using a constant temperature bath of LAUDA. The temperature in the cell was measured by CUSTOM’s Digital thermometer CUSTOM CT. A disk-shaped gel was cut by a glass tube with an inner diameter of 19 mm and placed in a cell filled with pure water to measure the degree of swelling. The temperature was kept at 20°C. using a constant temperature bath, and after allowing it to stand for one day, the diameter of the gel was compared with a disk-shaped silicone with 20 mm diameters. From 20°C to 60°C, the diameter of the gel at each temperature was observed over a sufficient time so that the gel was in an equilibrium swelling state while gradually increasing and decreasing the temperature.

### 2.16. Observation of time change of gel swelling degree with temperature jump and measurement of relaxation time and cooperative diffusion coefficient

Disk-shaped gels that had reached equilibrium swelling at a constant temperature were placed in cells set at different temperatures, and changes in the degree of swelling were observed with a smartphone. The diameter of the disk-shaped gel that entered the equilibrium swelling state at the temperature before the temperature jump (*d*_i_), the time change of the diameter of the cylindrical gel that was put into the cell at a different temperature (*d*_t_), and the diameter *d*_eq_ in the equilibrium swollen state after a temperature jump, the change in the diameter of the gel can be approximated with the following equation:

(2) $$\frac{d_{t}-d_{\mathrm{eq}}}{d_{i}-d_{\mathrm{eq}}}\approx \frac{6}{\pi^{2}}\exp\left(-\frac{t}{\tau }\right)$$

Experimentally, the following equation, which was obtained by taking the logarithms of both sides, is used:

(3) $$\log\frac{d_{t}-d_{\mathrm{eq}}}{d_{i}-d_{\mathrm{eq}}}\approx \log\frac{6}{\pi^{2}}-\frac{t}{\tau }$$

By plotting the values of the left-hand side of this equation against time τ and obtaining the relaxation time τ from the slope at a time when the intercept was fixed at $\log\frac{6}{\pi^{2}}$, a linear approximation can be applied. As a result, the collective diffusion coefficient *D* can be obtained for a disk-shaped and expressed by the following equation:

(4) $$D=\frac{{\left(\frac{d}{\pi }\right)}^{2}}{\tau }$$

### 2.17. DLS measurement of star-shaped PNIPA-b-PNEAA terminated with the azido group

To synthesize gels of a uniform network structure, creating a semidilute solution state in which the space within the solution is uniformly filled with terminal azide 4-armed star PNIPA-b-PNEAA is desirable. By measuring the scattering intensities from solutions of various volume fractions of terminal azide 4-armed star PNIPA-b-PNEAA by DLS, we estimated the volume fraction at which the transition of terminal azide 4-armed star PNIPA-b-PNEAA from dilute solution to semidilute solution occurs (volume fraction of overlap).

(5) $$\Phi \;=\frac{V_{p}}{V}\;=\frac{V_{p}}{V_{s}+V_{p}}=\frac{m_{p}{\overset{ˉ}{v}}_{p}}{m_{s}{\overset{ˉ}{v}}_{s}+m_{p}{\overset{ˉ}{v}}_{p}}$$

Where *V* is the total volume of solution, *m*_*s*_ and *m*_*p*_ are the mass of solvent and polymer, respectively. ${\overset{ˉ}{v}}_{s}$ is the solvent partial specific volume and ${\overset{ˉ}{v}}_{p}$ is the polymer’s partial specific volume.

Although the partial specific quantities are indeed functions of concentrations, it is often assumed – with some potential for error – that they are constants. Determining the partial specific volumes over the whole concentration range is a nuisance. Therefore,

(6) $$\rho_{s}=\mathrm{solvent}\;\;\mathrm{density}\left(\mathrm{approximately}{\left({\overset{ˉ}{v}}_{s}\right)}^{-1}\;\right)$$

(7) $$\rho_{p}=\mathrm{polymer}\;\;\mathrm{density}\left(\mathrm{approximately}{\left({\overset{ˉ}{v}}_{p}\right)}^{-1}\;\right)$$

In conclusion, the volume fraction of the polymer is given by:

(8) $$\Phi \;≅{\left(\frac{V_{s}m_{p}}{\rho_{p}}+1\right)}^{-1}$$

Scattering intensities obtained from various volume fractions of terminal azide 4-armed star PNIPA-b-PNEAA/acetonitrile solutions are plotted versus volume fraction. Scattering due to the solvent acetonitrile was subtracted. The overlap region transitions from dilute solution in good solvent (*I* ~ *Φ*^1^) to semidilute solution’s theoretical scaling law region (*I* ~ *Φ*^−1/4^). The volume fraction of overlap was *Φ* ≈0.174, with maximum scattering intensity.

## 3. Result and discussion

### 3.1. Synthesis of star-shaped PNIPA-b-PNEAA with azido-ended groups by one-pot reaction

**Figure 1.**
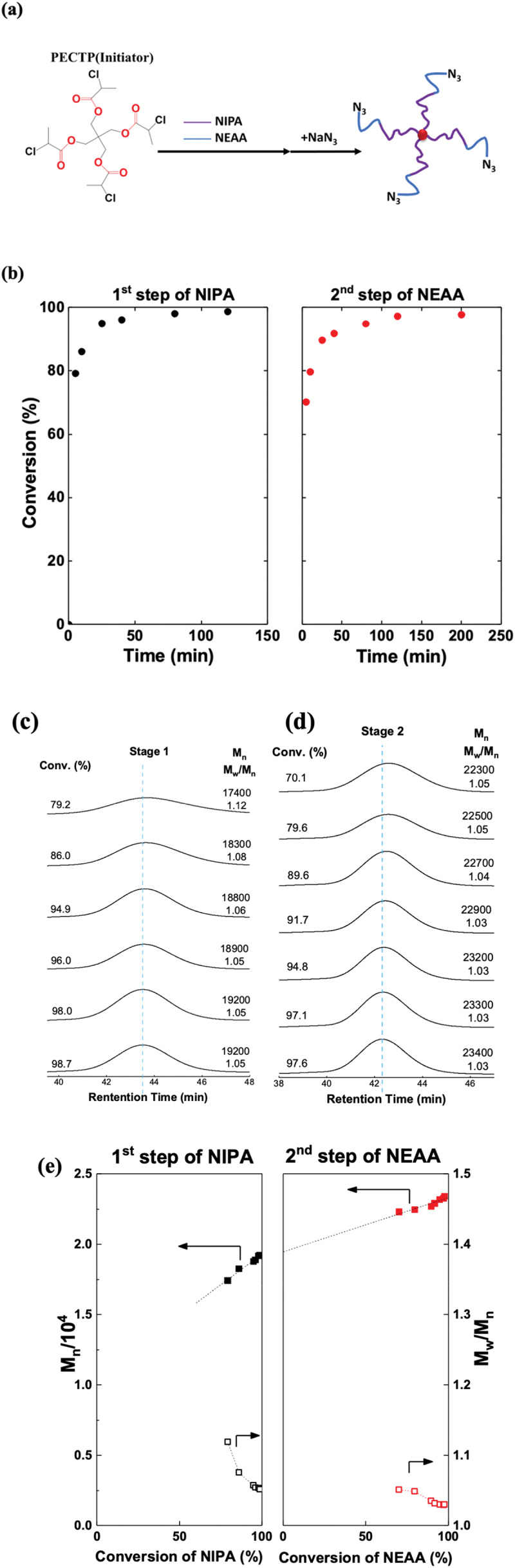
(a) Star polymer synthesis scheme comprising NIPA and NEAA. (b) Diagram plotting the monomer-to-polymer conversion versus reaction time during copper-catalyzed living radical polymerization of NIPA and NEAA. (c) Time evolution of SEC traces measured in copper-catalyzed living radical polymerization of NIPA. (d) Time evolution of SEC traces measured in copper-catalyzed living radical polymerization of NEAA. (e) Diagram showing the relationship between the number of average molecular weight, *M*_n_, and polydispersity index (*M*_w_/*M*_n_) of the obtained polymer and the conversion during copper-catalyzed living radical polymerization of NIPA and NEAA.

As a first step, star-shaped polymers consisting of NIPA were synthesized by polymerizing NIPA with the four-branched initiator pentaerythritol tetra(2-chloropropionate (PETCP), CuCl as catalyst, and tris[2-(dimethylamino)ethyl]amine (Me_6_TREN) as ligand in dimethylformamide (DMF)/H_2_O mixed solvent at 0°C (Figure 1(a)). From the ^1^H NMR spectra, the peak of the vinyl group of NIPA was attenuated as the polymerization progressed (Figure S2(a)). The result of the polymerization conversion was calculated from the vinyl group of NIPA, and the DMF aldehyde peak is shown in (Figure 1(b)). The reaction conversion reached 90% at approximately 30 min. In a second step, NEAA with mixed solvent DMF/H_2_O was immediately added to the polymerization system to synthesize block copolymer. From the ^1^H NMR spectra, the peak of the vinyl group of NEAA was attenuated as the polymerization progressed (Figure S2b). The result of the polymerization conversion was calculated from the vinyl group of NEAA, and the DMF aldehyde peak is shown in Figure 1(b). The reaction conversion reached above 90% at approximately 100 min. The results of the SEC measurement of four-branched star PNIPA and four-branched star PNIPA-b-PNEAA are shown in (Figure 1(c,d)). We elucidated that the molecular weight increased as the reaction proceeded. The graphs plot the average molecular weight (Mn) and molecular weight distribution (Mw/Mn) concerning the reaction conversion rate shown in Figure 1(e) for star-shaped PNIPA and star-shaped PNIPA-b-PNEAA, respectively. In this reaction system, the polymerization of NIPA progressed in a living manner, with a molecular weight of about 19,000 and a molecular weight distribution of 1.05 at a reaction rate of 98.7%. When NEAA was subsequently added, the polymerization of NEAA proceeded in the same living manner as that of NIPA, and at a reaction rate of 97.6%, the molecular weight was about 23,000, and the molecular weight distribution was 1.03. The SEC results showed no side reactions, such as dimer formation, during the progress of this polymerization.

The end group transformation from the chlorine group to the azide group of the four-branched star PNIPA-b-PNEAA directly proceeded immediately by adding excess sodium azide after the polymerization conversion reached between 90% and 95% without performing purification. A prerequisite to achieving high functionality by end group transformation is that the halogenated chain end is stable throughout the polymerization. When preparing block copolymers using acrylamide derivatives, the disappearance of the halogen end group is observed to be higher in tertiary acrylamides than in secondary acrylamides. This difference can be attributed to the electron density of the amide group, which is increased by increasing the alkyl substitution on the nitrogen atom. The higher electron density, influenced by inductive effects, promotes the cyclization reaction of the halogenated end group, leading to an enhanced termination rate and loss of active polymer chains. In the context of aqueous Cu-mediated polymerization of (meth)acrylamides, one of the reasons for the loss of control is the substitution of the terminal halogen with other functional groups, resulting in the formation of cyclic onium species. The copper complexes used in the polymerization process cannot effectively activate these species. As a result, the transformation or displacement reaction of the halogenated end group is hindered, leading to difficulties in controlling the polymerization and achieving the desired end group modifications. Various factors can influence the rate of displacement reaction [27–29]. Hence, NEAA was selected as a suitable monomer for the second block to reach high functionality.

The ^1^H NMR spectroscopy of the four-branched star PNIPA-b-PNEAA ended with the azido group, shown in (Figure S3). The clean formation of the azide-terminated PNIPA-b-PNEAA was evidenced by a peak (e) at approximately 3.6 ppm corresponding to the proton geminal to the azide group. In addition, the integral ratio of the peak (e) to the peak (c) derived from NIPA was e/c = 1: 40.2, which, compared to the ideal ratio e/c = 1: 40, indicated the completion of the end group transformation from chlorine to the azide.

### 3.2. Synthesis of star-shaped PNIPA-b-PNEAA with DBCO-amine ended group by strain-promoted azide-alkyne cycloaddition (SPAAC) click reaction

To quantitatively confirm the number of azide groups introduced at the ends of star-shaped polymers, the appropriate functional group was raised by a click reaction to the star-shaped polymers with azide groups introduced at the ends as described in the previous chapter as follows.

**Figure 2.**
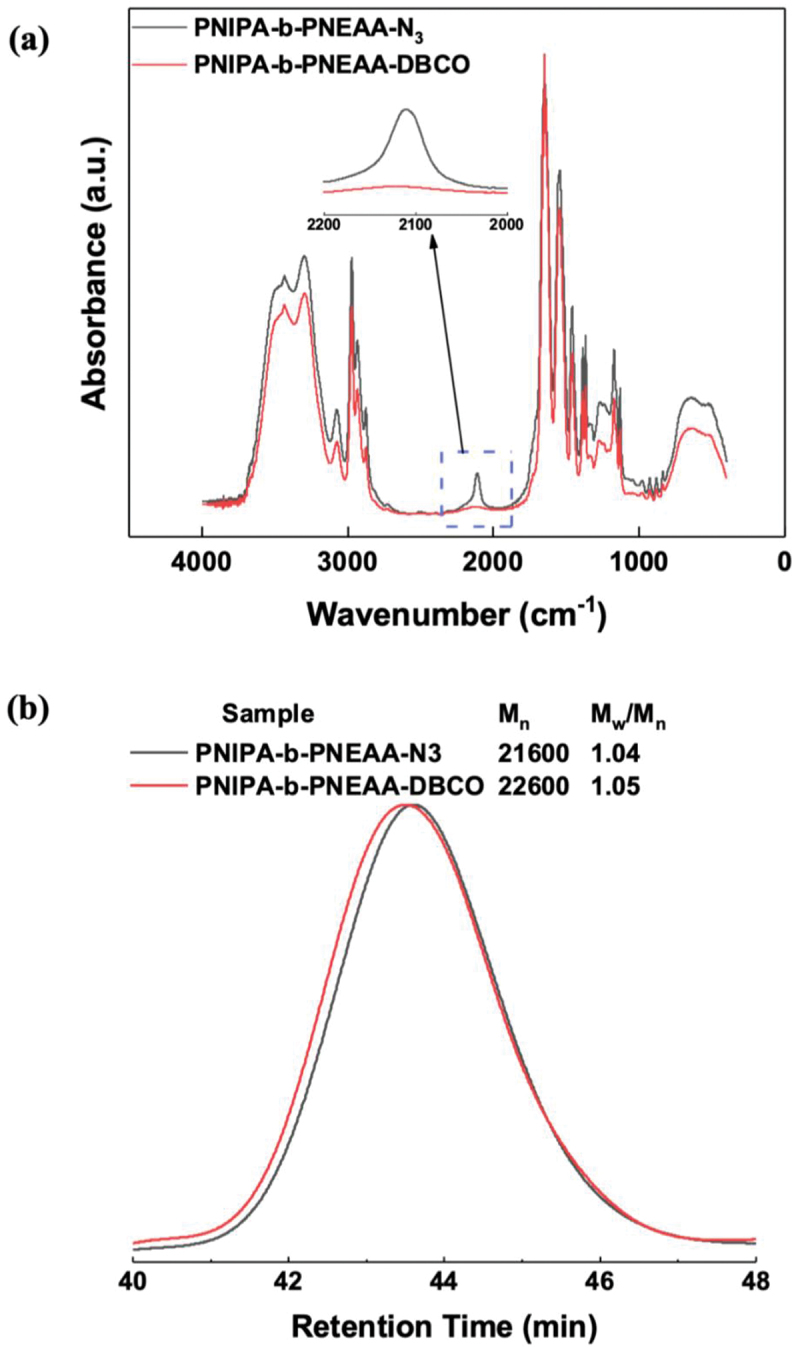
(a) ftir spectra of tetra-branched star PNIPA, obtained before and after SPAAC click reaction at room temperature. (b) Evolution of SEC traces of tetra-branched star PNIPA, obtained before and after SPAAC click reaction at room temperature.

The novel copper-free click chemistry is based on the reaction of a diaryl cyclooctene moiety with an azide-labeled reaction partner, known as strain-promoted alkyne azide cycloaddition (SPAAC). This new ‘Click reaction’, unlike conventional Click Chemistry, is very fast at room temperature and does not require a cytotoxic Cu(I) catalyst (that is toxic to most organisms) and, thus, prevents its use in many biological systems. Diarylcyclooctynes are thermostable with very narrow and specific reactivity toward azides, resulting in almost quantitative yields of stable triazoles. The strain-promoted or Cu(I)-free cycloaddition (SPAAC) strategy relies on strained cyclooctene. Diarylcyclooctynes are thermally stable compounds with very narrow and specific reactivity toward azides. Their use decreases the activation energy for the cycloaddition click reaction, enabling it to be carried out without the need for catalysis at low temperatures with an efficiency more significant than that of the Cu(I)-catalyzed ligation. The FTIR measurement results before and after the click reaction are shown in Figure 2(a). The terminated azide groups’ peak (2100 cm^−1^) disappeared completely after the click reaction. From this result, we can conclude that the click reaction progressed. The ^1^H NMR spectroscopy of the four-branched star PNIPA ended with the dibenzocyclooctyne-amine (DBCO-amine) group is shown in (Figure S4). The clean formation of the four-branched star PNIPA ended with the DBCO-amine group, evidenced by the peaks (e) at approximately 7.2–7.6 ppm corresponding to the protons on the benzyl groups. In addition, the integral ratio of the peak (e) to the peak (c) derived from NIPA was c/e = 4.8, which, compared to the ideal ratio c/e = 5, indicated the completion of the SPAAC click reaction. The results of the SEC measurement of the four-branched modified star PNIPA are shown in Figure 2(b). The number-average molecular weight changes reveal that the SPAAC click reaction was completed because the changes are approximately four times the DBCO-Amine’s molecular weight. On the other hand, the experiment could strongly support the completion of the end group transformation from the chlorine to the azide group.

### 3.3. Methanolysis degradation of star-shaped PNIPA-b-PNEAA terminated with the azido group

**Figure 3.**
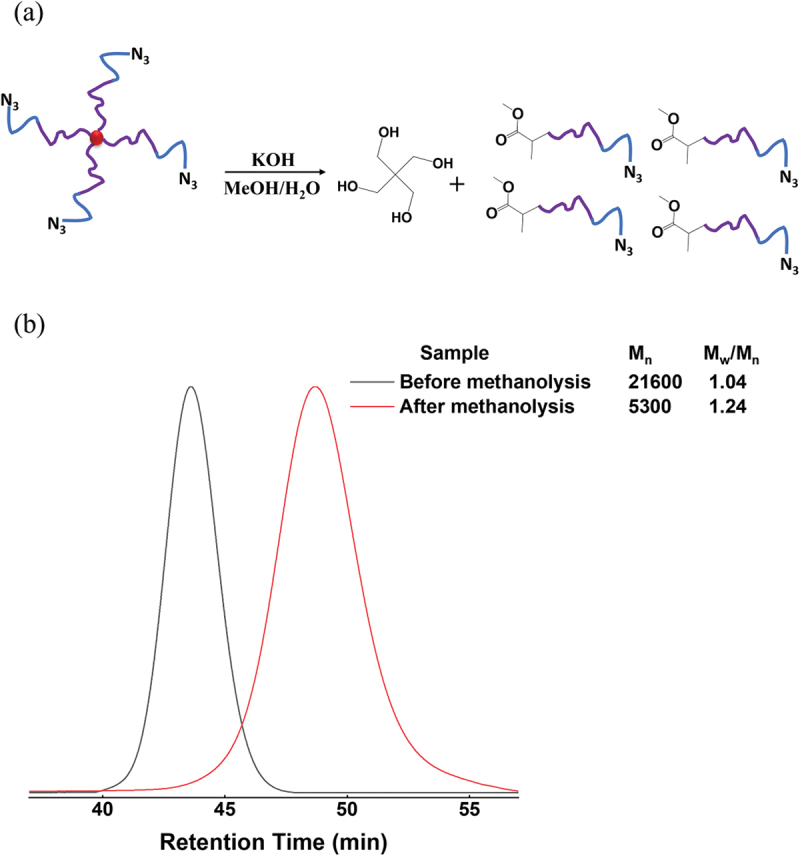
(a) Scheme of methanolysis of star-shaped PNIPA-b-PNEAA with azido-ended groups, (b) Evolution of SEC traces with methanolysis of PNIPA-b-PNEAA ended with the azide groups.

As shown in Figure 1(a), the initiator PETCP has four initiating groups bonded via ester groups. To confirm that the chains are equally extended from the four initiating groups of the initiator, the terminal azide 4-armed star PNIPA-b-PNEAA was subjected to methanolysis degradation at the initiator moiety Figure 3(a). The structure of terminal azide 4-armed star PNIPA-b-PNEAA was analyzed by SEC measurement of the resulting degradation products. Figure 3(b) shows the results of SEC measurements of the polymer before and after methanolysis degradation of terminal azide 4-armed star PNIPA-b-PNEAA. The molecular weight distribution of the polymer obtained by methanolysis was narrow (1.24), and the average molecular weight was about a quarter of the molecular weight of the polymer before methanolysis. Therefore, it was concluded that the desired star-shaped polymer, consisting of four connected polymers of similar molecular weight, could be synthesized. We confirmed that we had synthesized a terminal azide 4-armed star PNIPA-b-PNEAA from the above.

### 3.4. Preparation of PNIPA-b-PNEAA gels by double strain-promoted azide-alkyne cycloaddition via bimolecular homodifunctional method

**Figure 4.**
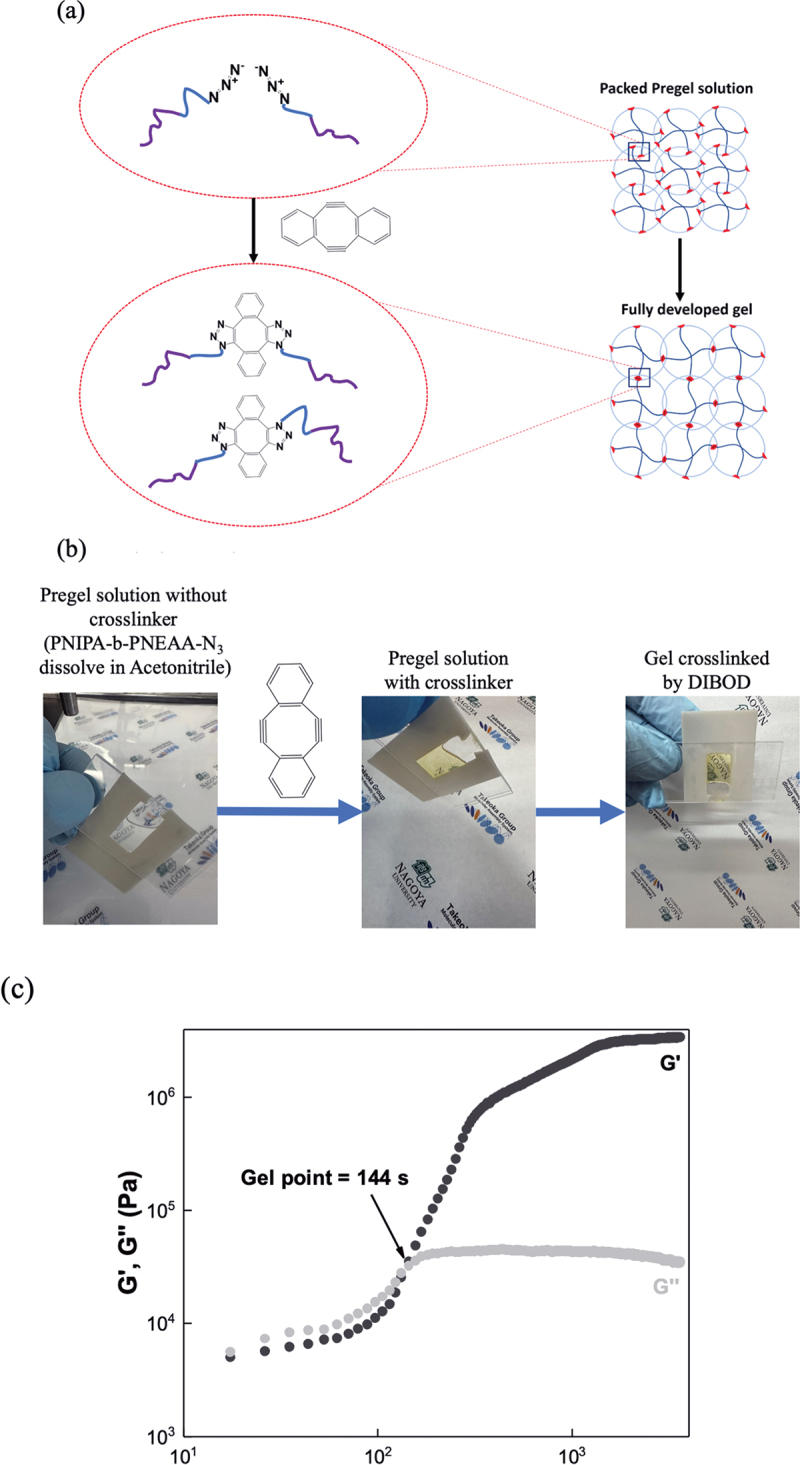
(a) Conceptual diagram of the formation of a polymer network using a click reaction under semidilute solution conditions in a good solvent for star-shaped polymers., (b) Photographic images of star-PNIPA-b-PNEAA solutions before and after gelation, (c) Gelation of the star polymers is confirmed by dynamic mechanical analysis. *G*′ and *G*″ represent the storage and loss shear moduli, respectively.

Monodisperse star polymers with multiple arms exhibit a strong excluded volume effect in good solvents, preventing other polymers from entering the percolated volume. Our star polymers were dissolved in a good solvent at a concentration well above the star polymer chain overlap concentration (Φ*) to ensure that the star polymers occupied the space uniformly and tightly, as assumed by the bond percolation model Figure 4(a). While only a single decay appears in the PNIPA-b-PNEAA/acetonitrile solution, an additional slow decay appears in the PNIPA-b-PNEAA/DMF and PNIPA-b-PNEAA/THF solution, suggesting the segregation of the PNIPA-b-PNEAA chains in DMF and THF. Anhydrous acetonitrile was chosen as a good solvent to prevent segregation of the polymer chains during crosslinking (Figure S5). Then, we estimated the value of Φ* of our star-shaped polymers in acetonitrile solution by examining the dependence of the light scattering intensity on the polymer concentration (Figure S6).

As a result of adding the crosslinking agent, sym-dibenzo-1,5-cyclooctadiene-3,7-diyne (DIBOD), to the solution, gelation proceeded in several minutes to obtain a diblock copolymer gel (Figure 4(a,b)). We confirmed the gelation using dynamic mechanical analysis. When the gelation reaction was carried out above the overlapping concentration (*Φ* = 1.75Φ*), the shear-storage moduli (*G*′) overtook the shear-loss moduli (*G*″) during the gelation process, indicating the successful formation of a gel network Figure 4(c). After the gelation proceeded sufficiently, the sample was removed and put into THF to wash out the unreacted material. The gel was put into methanol and water for 2–3 days for a solvent exchange.

### 3.5. Methanolysis degradation of gels

**Figure 5.**
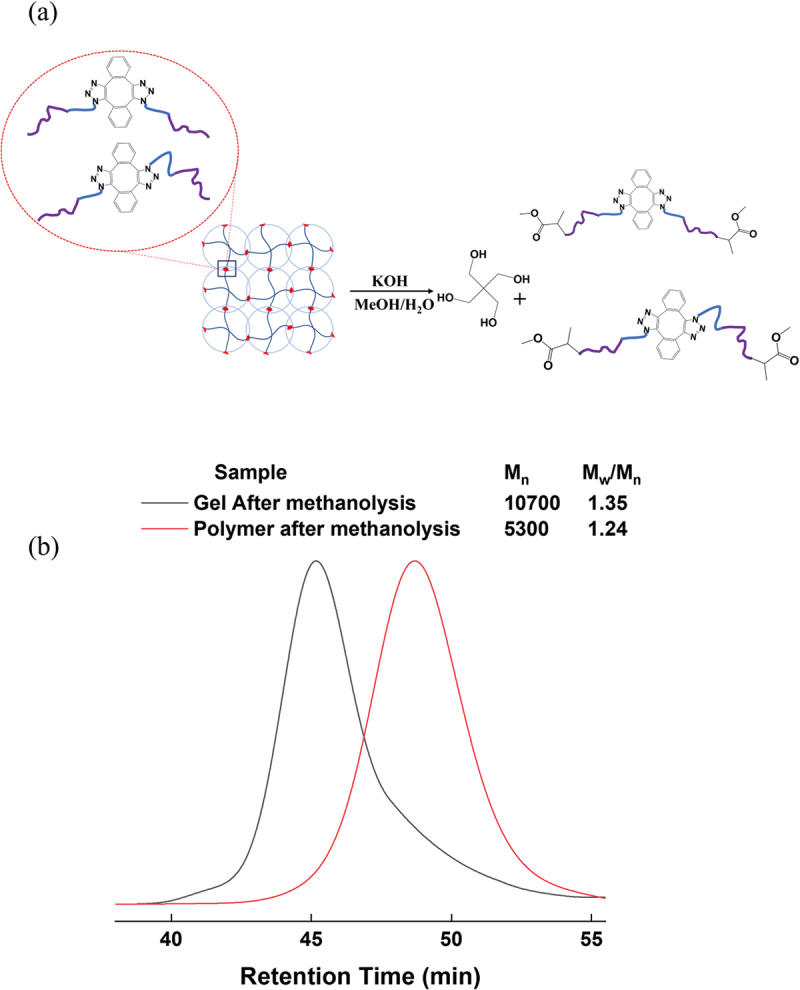
(a) Scheme of methanolysis of PNIPA-b-PNEAA gel, (b) Evolution of SEC traces with methanolysis of gel crosslinked by DIBOD.

The polymer network of the gel underwent methanolysis degradation, and the original polymer network’s structure was evaluated by analyzing degradation products Figure 5(a). An inductive assessment was employed. If all azido groups reacted with DIBOD to achieve a relatively homogeneous network structure, the polymer’s average molecular weight after methanolysis would be twice that of the degraded star-shaped PNIPA-b-PNEAA. Conversely, if the azide group remained unreacted, the polymer’s average molecular weight after methanolysis degradation would match the average molecular weight of the methanolysis fraction of star-shaped PNIPA-b-PNEAA. Thus, the analysis of the polymer solution obtained through methanolysis degradation of the gel, using SEC measurement, enables the examination of the uniformity of the original network structure.

The SEC measurement results of the gel synthesized under a volume fraction of 1.75 Φ*, which is sufficiently higher in concentration than the overlapping concentration Φ* of the star-shaped polymer, after methanolysis gel degradation, are shown in Figure 5(b). Although there is a slight deformation of the SEC curve towards the long-time side due to the presence of non-dimerized polymer chains, *M*_w_/*M*_n_ of the polymer obtained by methanolysis is narrow (1.35), and *M*_n_ of the polymer obtained by methanolysis was about twice the average molecular weight of the polymers obtained by degradation of star-shaped PNIPA-b-PNEAA. These results confirm that the gels obtained after crosslinking with DIBOD have few uncross-linked chains, uniform molecular weight between crosslinking points, and few dangling chains. In our previous paper [12], we synthesized two types of polymers, one star-shaped polymer with an amino group at the end and the other with a carboxyl group at the end, and used these neutralization reactions to dimerize the polymers efficiently. However, under semidilute conditions of star-shaped polymers, we found that the click reaction can be used for very efficient polymer dimerization.

### 3.6. Observation of the swelling and shrinking behavior of thermo-responsive polymer gels

**Figure 6.**
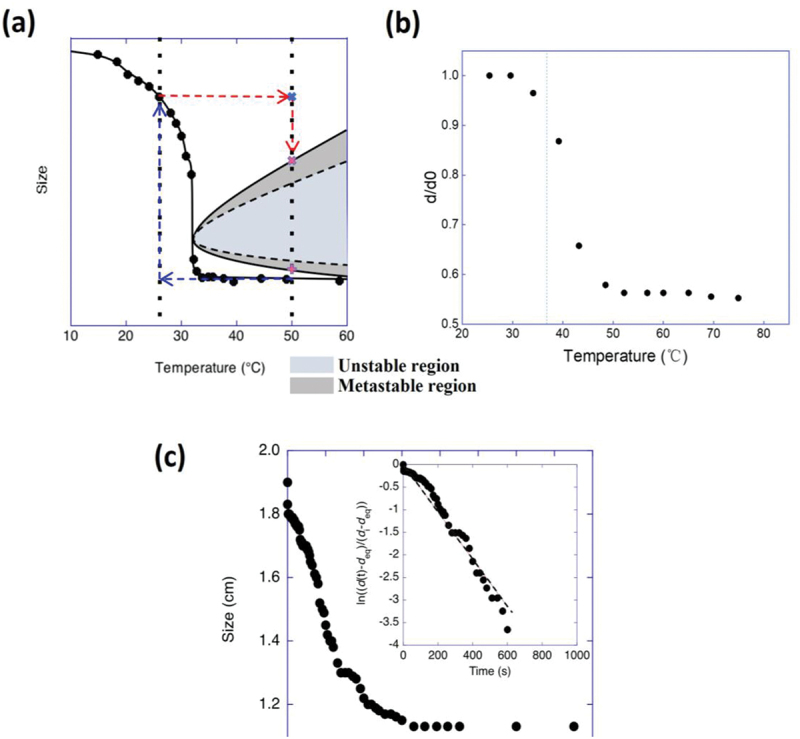
(a) Schematic diagram showing the temperature-dependent phase diagram of the swelling degree of the polymer gel consisting of NIPA. (b) Temperature dependence of the degree of swelling in water of the PNIPA-b-PNEAA polymer gel. (c) Kinetic change in the size of the PNIPA-b-PNEAA gel by temperature jump from 20°C to 60°C. The change in the diameter of the disk-shaped gel over time was observed. The thickness of the disk-shaped gel is 2.13 mm at 20°C.

Gels consisting solely of NIPA generally exhibit a slow volume contraction in response to a rapid temperature rise across the LCST. This phenomenon is mainly due to the rapid formation of a skin layer on the surface of gels exposed to high-temperature environments and the phase separation of the polymer network inside the gel as the gel reaches an unstable region Figure 6(a) [30–32]. As these phenomena do not occur in the volume swelling of the gel caused by a rapid temperature drop across the LCST, the relaxation time (τ) required for volume change is elucidated by the following equation derived from Tanaka-Fillmore’s theory. This equation utilizes the characteristic length (*l*) of the polymer gel (e.g. the radius of a spherical polymer gel) and the collective diffusion coefficient of the three-dimensional polymer network (*D*) [33,34].

(9) $$\tau ∼\frac{l^{2}}{D}$$

Even when phenomena like skin layer formation or macrophase separation occur due to volume changes, the time variation of gel size is continuous. The analysis using this Equation (9) can determine the value of *D* affected by such phenomena. Given that τ is impacted by the size and shape of the gel, the value of *D* is employed here to explore the correlation with the network structure and the swelling/contraction behavior of each gel.

The temperature dependence of the equilibrium swelling of PNIPA-b-PNEAA gels is shown in Figure 6(b). The phase transition temperature of PNIPA is about 32°C. In contrast, the phase transition of PNEAA occurs at about 82°C because the ethyl groups on the nitrogen atoms are more hydrophilic than the isopropyl groups in the NIPA monomer. Due to the block copolymerization of PNIPA and PNEAA, our gels consisting of PNIPA-b-PNEAA star-shaped polymers exhibit a temperature change in volume of about 37°C.

The time course of the volume change associated with the shrinkage of the gel was observed by a temperature jump from 20°C to 60°C, followed by a rapid change in the degree of swelling of the gel. The time course of gel size is shown in Figure 6(c). As seen from Figure 6(c), the outer radius shift was fast at first but slowed down. This phenomenon may be due to the formation of a skin layer and phase separation, but the shrinkage change was smoother than in the case of conventional gels consisting only of NIPA; the effect of incorporating the polymer chains of NEAA into the polymer network as a block copolymer may be responsible for the smooth shrinkage change. Based on Figure 6(c), an inset of Figure 6(c) was prepared, and the gel’s collective diffusion coefficient of the polymer network was calculated assuming a series of changes in the plot over time, which was 7.81 × 10^−6^ cm^2^/s. We know that the collective diffusion coefficient of conventional NIPA gels during shrinkage is around 1 × 10^−9^ to 1 × 10^−8^ cm^2^/s. The value of the collective diffusion coefficient observed in this study is more than an order of magnitude greater than those of conventional NIPA gels at shrinkage and comparable to those at swelling.

## 4. Conclusion

An efficient method has been devised for preparing terminal azide 4-branched star PNIPA-b-PNEAA, characterized by a narrow molecular weight distribution and requisite molecular weight for successful synthesis. A thermo-responsive polymer gel featuring a controllable network structure was also produced by dimerizing azide-terminated polymers through the self-accelerating DSPAAC click reaction. This approach is convenient as azide-terminated polymers can be easily prepared through post-modification techniques. The self-accelerating nature of DSPAAC obviates the need for equimolar amounts of complementary reaction groups in the gelation process. Moreover, the periodic introduction of temperature-independent hydrophilic polymer chains into the network allows for smooth volume contraction of the gel. By leveraging the distinct advantages of metal-free, mild reaction conditions, and high efficiency, the DSPAAC-based polymer crosslinking method holds promise as a potent tool for creating topological polymers with a symmetric molecular structure. By achieving a uniform network structure in various functional gels, the development of novel functional polymer gels is foreseeable, showcasing advancements in mechanical properties, optical properties, substance separation capability, substance adsorption ability, and substance release capability.

## Supplementary Material

Supplemental Material

## References

[1] Tanaka M, Nakahata M, Linke P, et al. Stimuli-responsive hydrogels as a model of the dynamic cellular microenvironment. Polym J. 2020;52(8):861–15. doi: 10.1038/s41428-020-0353-6

[2] Knipe JM, Peppas NA. Multi-responsive hydrogels for drug delivery and tissue engineering applications. Regener Biomater. 2014;1(1):57–65. doi: 10.1093/rb/rbu006 PMC4669007 26816625

[3] Bin Imran A, Seki T, Takeoka Y. Recent advances in hydrogels in terms of fast stimuli responsiveness and superior mechanical performance. Polym J. 2010;42(11):839–851. doi: 10.1038/pj.2010.87

[4] Osada Y, Okuzaki H, Hori H. A polymer gel with electrically driven motility. Nature. 1992;355(6357):242–244. doi: 10.1038/355242a0

[5] Wang GQ, Kuroda K, Enoki T, et al. Gel catalysts that switch on and off. Proc Natl Acad Sci, USA. 2000;97(18):9861–9864. doi: 10.1073/pnas.180192597 10954747 PMC27606

[6] Nakayama D, Takeoka Y, Watanabe M, et al. Simple and precise preparation of a porous gel for a colorimetric glucose sensor by a templating technique. Angew Chem Int Ed. 2003;42(35):4197–4200. doi: 10.1002/anie.200351746 14502734

[7] Holtz JH, Holtz JSW, Munro CH, et al. Intelligent polymerized crystalline colloidal arrays: novel chemical sensor materials. Anal Chem. 1998;70(4):780–791. doi: 10.1021/ac970853i

[8] Wang XH, Qiu XP, Wu C. Comparison of the coil-to-globule and the globule-to-coil transitions of a single poly(N-isopropylacrylamide) homopolymer chain in water. Macromolecules. 1998;31(9):2972–2976. doi: 10.1021/ma971873p

[9] Norioka C, Kawamura A, Miyata T. Relatively homogeneous network structures of temperature-responsive gels synthesized via atom transfer radical polymerization. Soft Matter. 2023;19(14):2505–2513. doi: 10.1039/D3SM00044C 36843532

[10] Norioka C, Kawamura A, Miyata T. Mechanical and responsive properties of temperature-responsive gels prepared via atom transfer radical polymerization. Polym Chem UK. 2017;8(39):6050–6057. doi: 10.1039/C7PY01323J

[11] Matyjaszewski K. Advanced materials by atom transfer radical polymerization. Adv Mater. 2018;30(23):1706441. doi: 10.1002/adma.201706441 29582478

[12] Okaya Y, Jochi Y, Seki T, et al. Precise synthesis of a homogeneous thermoresponsive polymer network composed of four-branched star polymers with a narrow molecular weight distribution. Macromolecules. 2020;53(1):374–386. doi: 10.1021/acs.macromol.9b01616

[13] Jochi Y, Seki T, Soejima T, et al. Spontaneous synthesis of a homogeneous thermoresponsive polymer network composed of polymers with a narrow molecular weight distribution. Npg Asia Mater. 2018;10(8):840–848. doi: 10.1038/s41427-018-0074-x

[14] Huang J, Kong SX, Tang ZH, et al. Facile strategy for the biomimetic heterogeneous design of elastomers with mechanical robustness, malleability, and functionality. ACS Macro Lett. 2020;9(1):49–55. doi: 10.1021/acsmacrolett.9b00845 35638670

[15] Sheiko SS, Dobrynin AV. Architectural code for rubber elasticity: from supersoft to superfirm materials. Macromolecules. 2019;52(20):7531–7546. doi: 10.1021/acs.macromol.9b01127

[16] Ida S, Toda S, Oyama M, et al. Multiarm star-crosslinked hydrogel: polymer network with thermoresponsive free-end chains densely connected to crosslinking points. Macromol Rapid Commun. 2020;42(8):2000558. doi: 10.1002/marc.202000558 33244811

[17] Vamvakaki M, Patrickios CS, Lindner P, et al. Amphiphilic networks based on cross-linked star polymers: a small-angle neutron scattering study. Langmuir. 2007;23(21):10433–10437. doi: 10.1021/la700933p 17824716

[18] Satoh K, Ozawa S, Mizutani M, et al. Sequence-regulated vinyl copolymers by metal-catalysed step-growth radical polymerization. Nat Commun. 2010;1(1):2149. doi: 10.1038/ncomms1004 20975670

[19] Badi N, Lutz JF. Sequence control in polymer synthesis. Chem Soc Rev. 2009;38(12):3383–3390. doi: 10.1039/b806413j 20449057

[20] Sun P, Chen J, Liu J, et al. Self-accelerating click reaction for cyclic polymer. Macromolecules. 2017;50(4):1463–1472. doi: 10.1021/acs.macromol.6b02614

[21] Liu X, Wu Y, Zhang M, et al. Efficient polymer dimerization method based on self-accelerating click reaction. RSC Adv. 2020;10(12):6794–6800. doi: 10.1039/C9RA09919K 35493909 PMC9049738

[22] Rosen BM, Percec V. Single-electron transfer and single-electron transfer degenerative chain transfer living radical polymerization. Chem Rev. 2009;109(11):5069–5119. doi: 10.1021/cr900024j 19817375

[23] Lligadas G, Grama S, Percec V. Single-electron transfer living radical polymerization platform to practice, develop, and invent. Biomacromolecules. 2017;18(10):2981–3008. doi: 10.1021/acs.biomac.7b01131 28849905

[24] Zhang Q, Wilson P, Li Z, et al. Aqueous copper-mediated living polymerization: Exploiting rapid disproportionation of CuBr with Me_6_TREN. J Am Chem Soc. 2013;135(19):7355–7363. doi: 10.1021/ja4026402 23597244

[25] Tang Q, Zhang K. Polymer synthesis based on self-accelerating 1, 3-dipolar cycloaddition click reactions †. Chin J Chem. 2021;39(11):3093–3100. doi: 10.1002/cjoc.202100305

[26] Kii I, Shiraishi A, Hiramatsu T, et al. Starin-promoted double-click reaction for chemical modification of azido-biomolecuels. Org Biomol Chem. 2010;8(18):4051–5055. doi: 10.1039/c0ob00003e 20657923

[27] Coessens V, Pintauer T, Matyjaszewski K. Functional polymers by atom transfer radical polymerization. Prog Polym Sci. 2001;26(3):337–377. doi: 10.1016/S0079-6700(01)00003-X

[28] Alsubaie F, Anastasaki A, Wilson P, et al. Sequence-controlled multi-block copolymerization of acrylamides via aqueous SET-LRP at 0 °C. Polym Chem. 2015;6(3):406–417. doi: 10.1039/C4PY01066C

[29] Wang CG, Goto A. Solvent-selective reactions of alkyl iodide with sodium azide for radical generation and azide substitution and their application to one-pot synthesis of chain-end-functionalized polymers. J Am Chem Soc. 2017;139(30):10551–10560. doi: 10.1021/jacs.7b05879 28741356

[30] Li Y, Tanaka T. Kinetics of swelling and shrinking of gels. J Chem Phys. 1990;92(2):1365–1371. doi: 10.1063/1.458148

[31] Takata S, Suzuki K, Norisuye T, et al. Dependence of shrinking kinetics of poly(N-isopropylacrylamide) gels on preparation temperature. Polymer. 2002;43(10):3101–3107. doi: 10.1016/S0032-3861(02)00089-7

[32] Takahashi K, Takigawa T, Masuda T. Swelling and deswelling kinetics of poly(N-isopropylacrylamide) gels. J Chem Phys. 2004;120(6):2972–2979. doi: 10.1063/1.1636695 15268444

[33] Tanaka T, Fillmore DJ. Kinetics of swelling of gels. J Chem Phys. 1979;70(3):1214–1218. doi: 10.1063/1.437602

[34] Nosaka S, Urayama K, Takigawa T. Static and dynamic swelling properties of poly(N-isopropylacrylamide) gels in the swollen state. Polym J. 2005;37(9):694–699. doi: 10.1295/polymj.37.694

