# Supplementary material for: Synthesis of thermo-responsive polymer gels composed of star-shaped block copolymers by copper-catalyzed living radical polymerization and click reaction
Source: Sci Technol Adv Mater. 2024 Feb 14;25(1):2302795. doi: 10.1080/14686996.2024.2302795 (PMC10868426; doi:10.1080/14686996.2024.2302795)
Supplement: Supplemental Material [file TSTA_A_2302795_SM4069.docx]

**Supporting Information**

**Synthesis of thermo-responsive polymer gels composed of star-shaped block copolymers by copper-catalyzed living radical polymerization and click reaction**

Guohao Gao^1^, Mitsuo Hara^1^, Takahiro Seki^1^, and Yukikazu Takeoka^1,*^

^1^*Department of Molecular & Macromolecular Chemistry, Nagoya University, Nagoya 464-8603, Japan*

Email: [ytakeoka@chembio.nagoya-u.ac.jp](mailto:ytakeoka@chembio.nagoya-u.ac.jp), Phone number: +81-52-789-4670


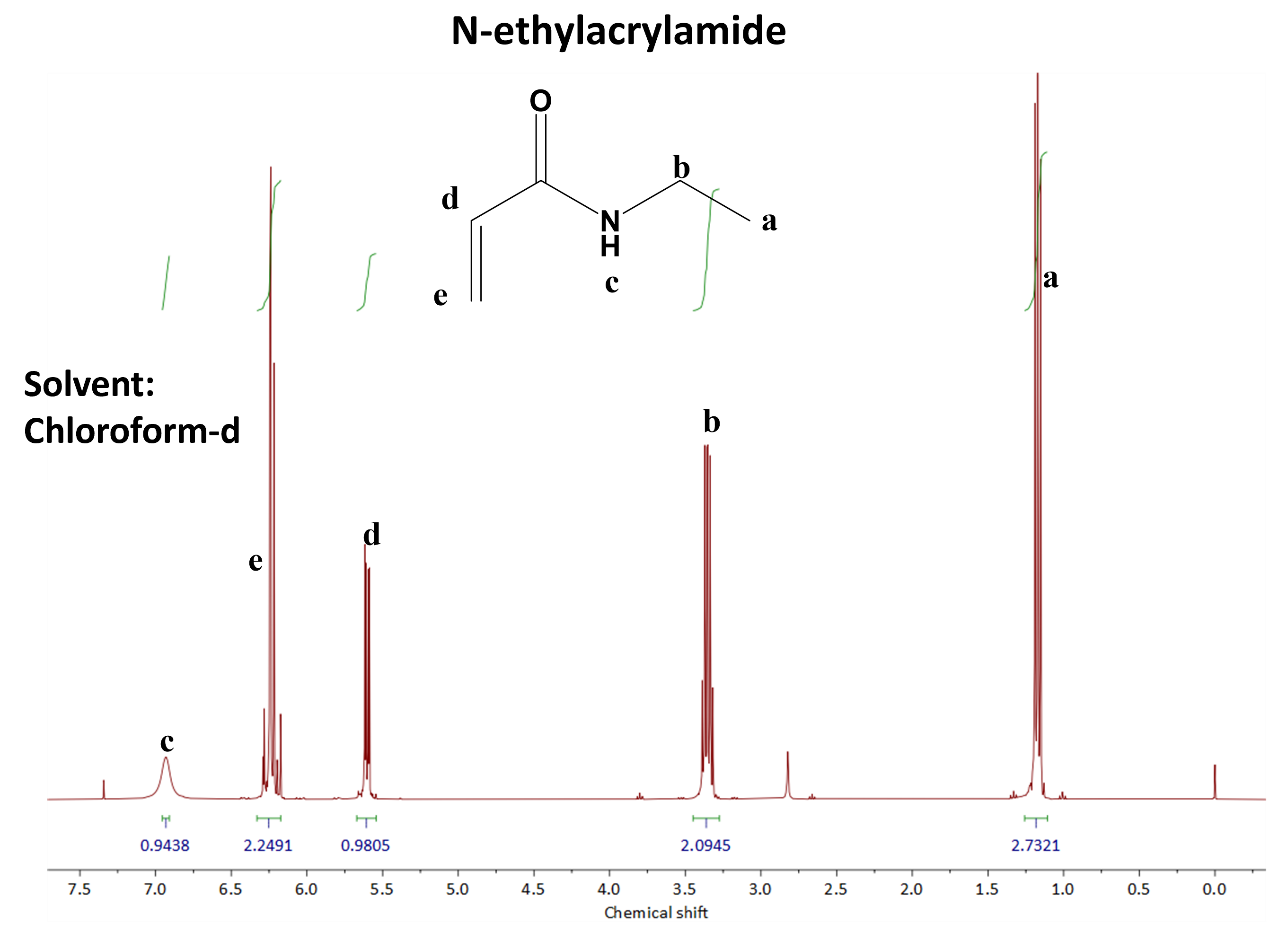


Fig. S1. ^1^H NMR spectrum of N-ethyl acrylamide


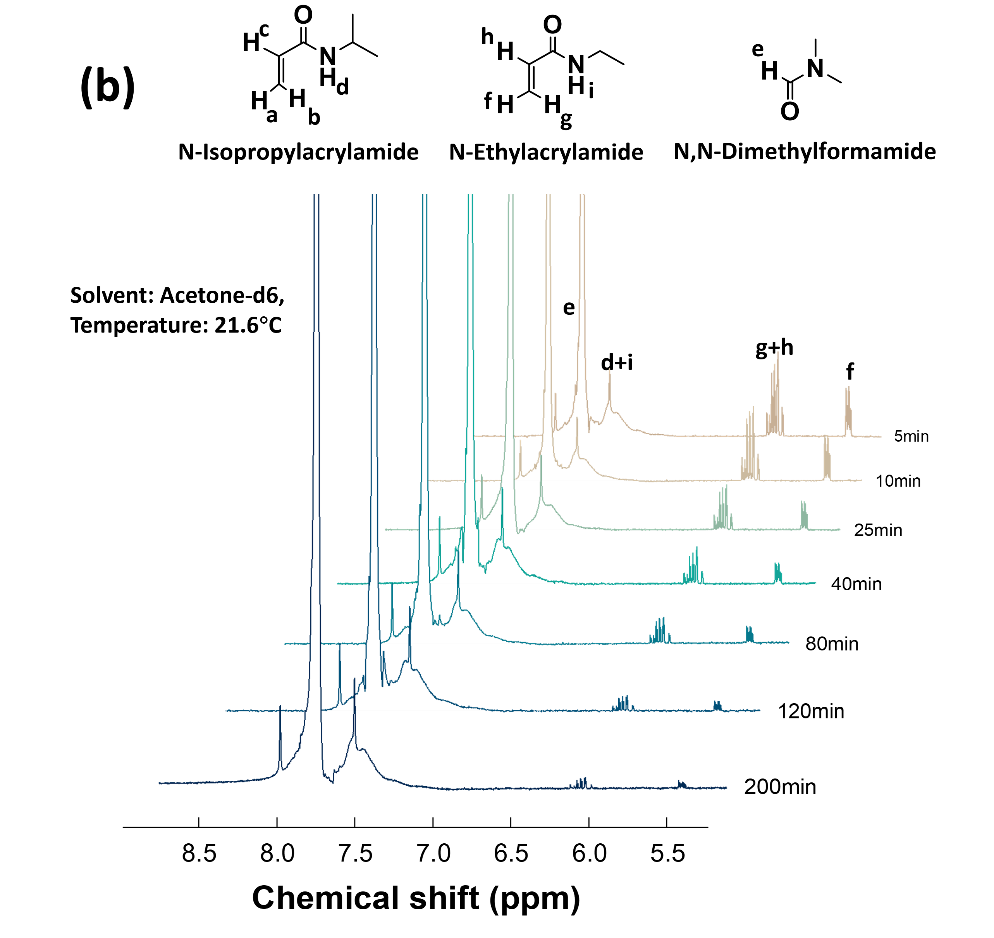

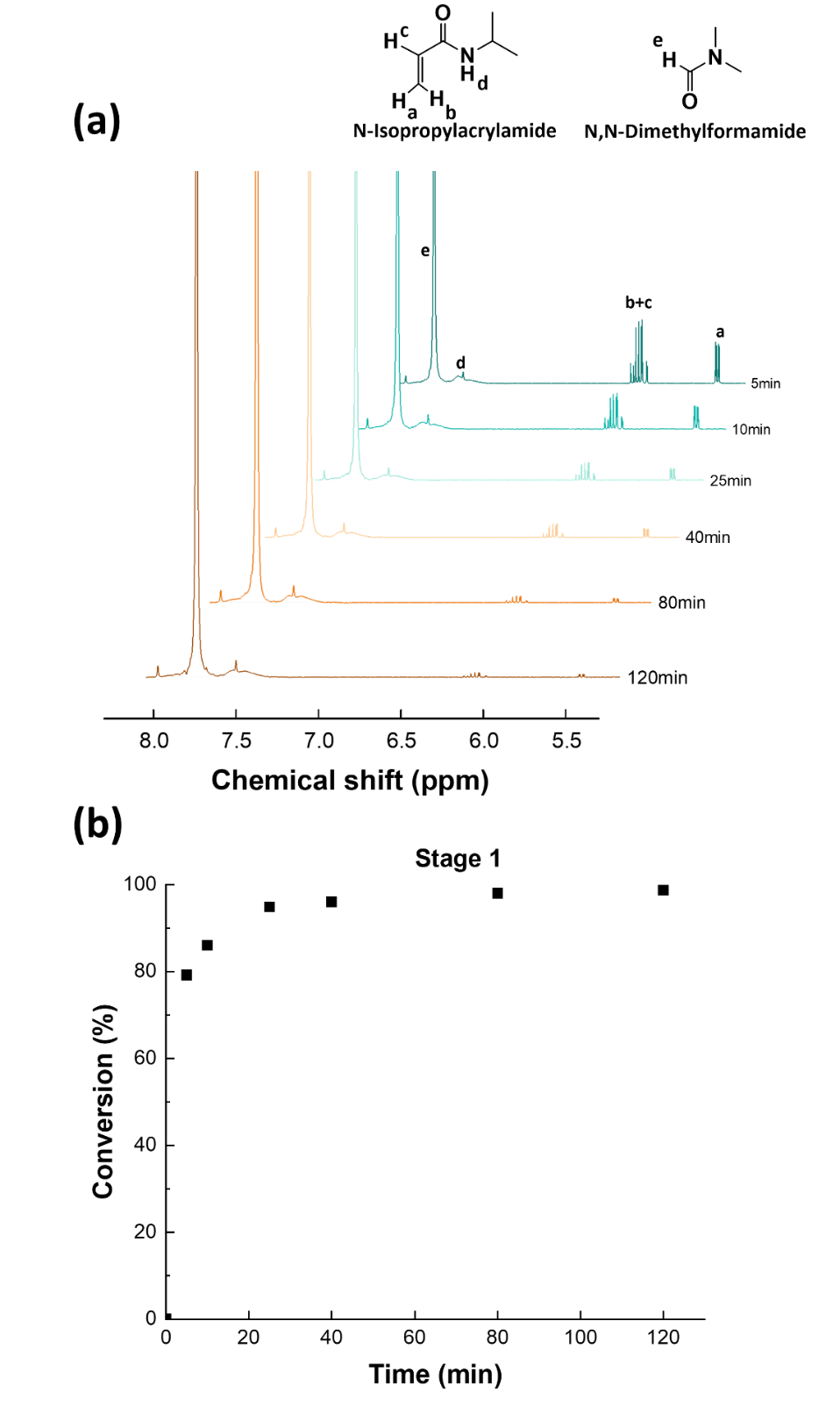


Fig. S2(a). Results of time variation of ^1^H NMR spectra for the reaction of a four-branched initiator with NIPA(stage 1). (b) Results of time variation of ^1^H NMR spectra for the reaction of a four-branched initiator with NEAA.


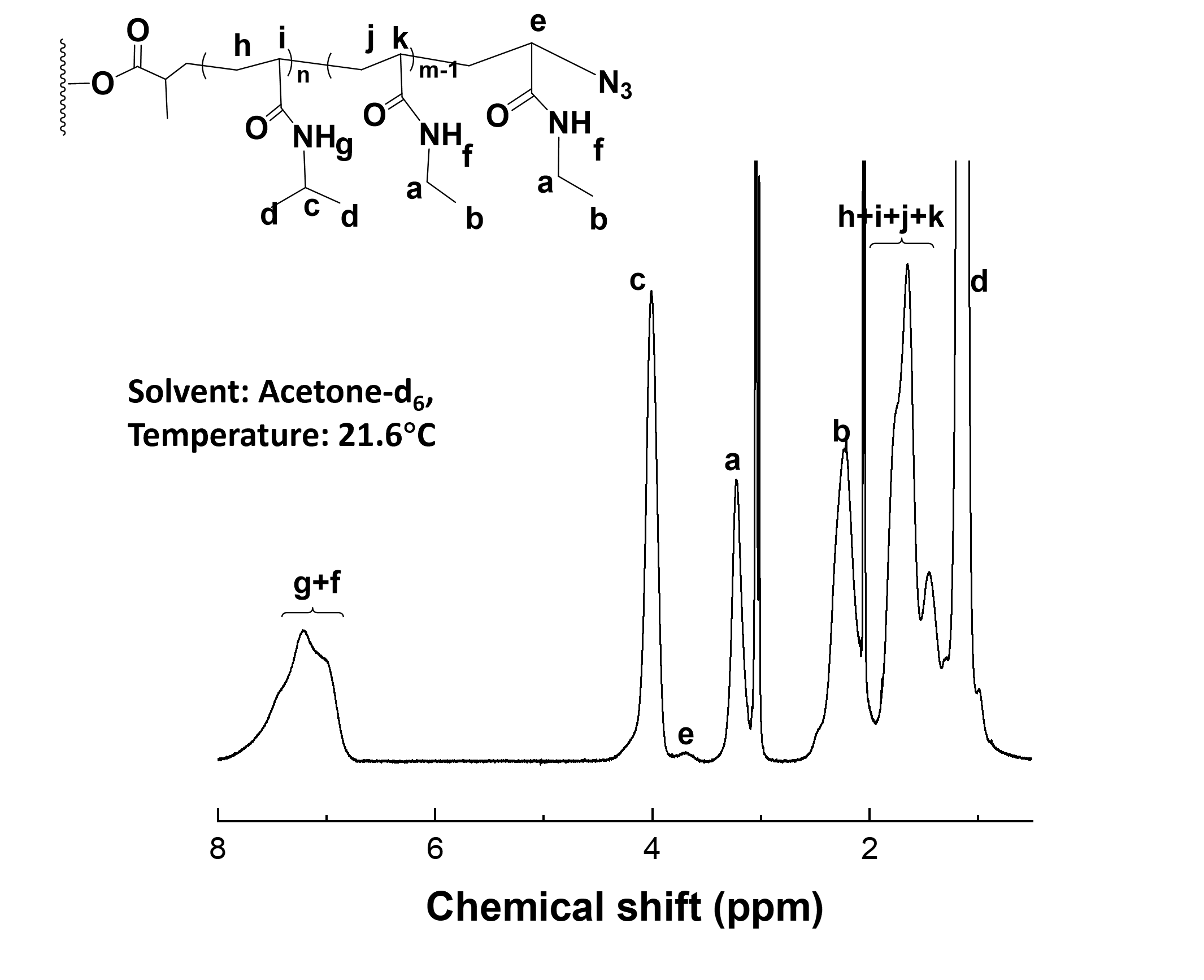


Fig. S3 ^1^H NMR spectroscopy of Azide-terminated tetra-branched star PNIPA prepared by one-pot synthesis.


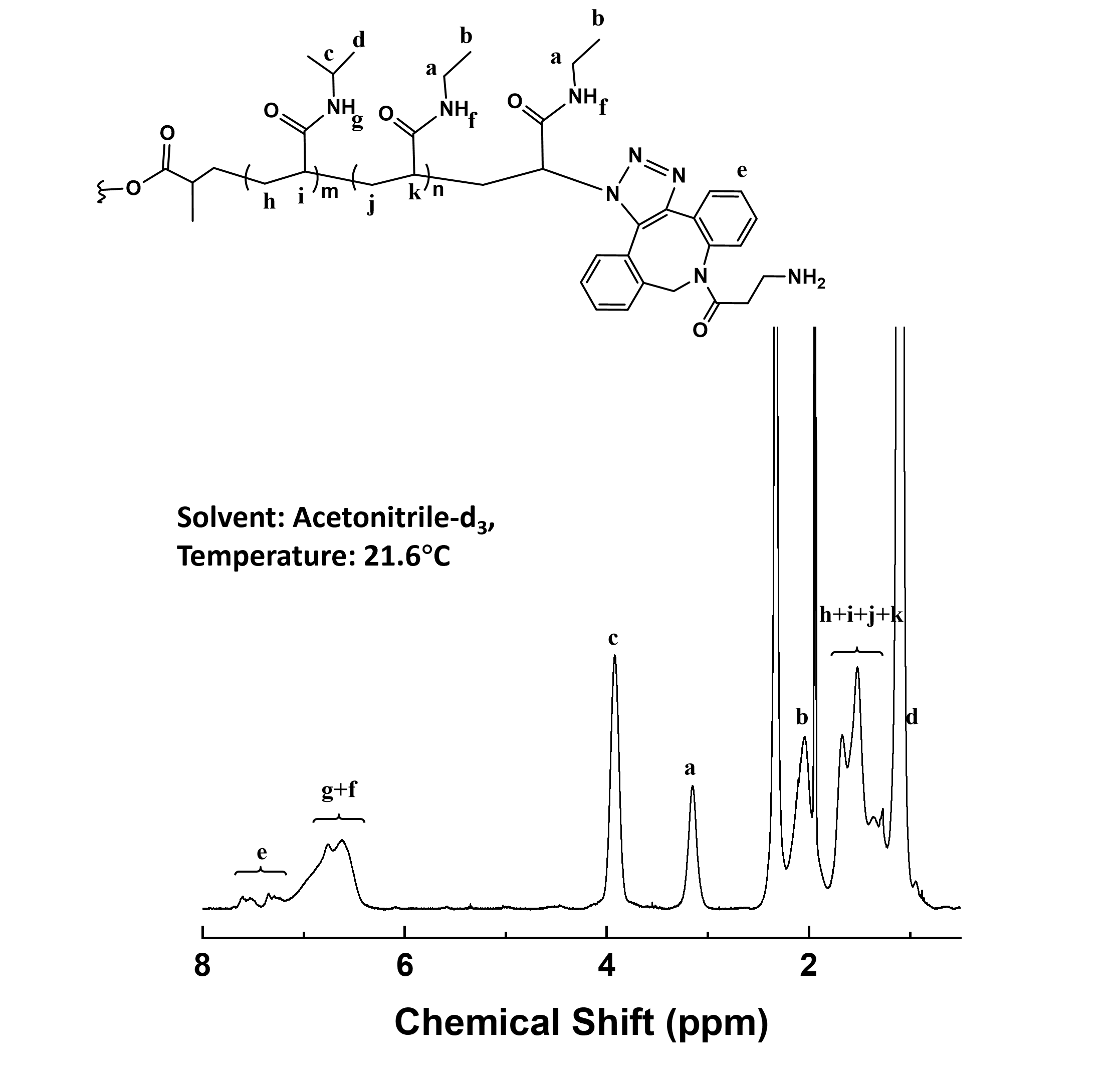


Fig. S4 ^1^H NMR spectroscopy of tetra-branched star PNIPA-b-PNEAA teminated with DBCO-amine prepared by SPAAC click reaction.


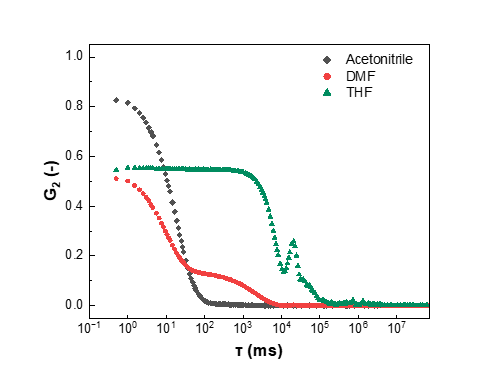


Fig. S5 Time correlation function (*g*_2_) of the star PNIPA-b-PNEAA-N_3_ in acetonitrile, DMF, and THF without the addition of the cross-linker. The star polymer concentration in each solution is Φ*, the same as that in Fig. S4. All solutions are mixed thoroughly with a centrifuge mixer and then filtered through general syringe filters with a pore size of 0.45 μm to remove dust and any impurities before the dynamic light scattering measurements. While only a single decay appears in the PNIPA-b-PNEAA/acetonitrile solution, an additional slow decay appears in the PNIPA-b-PNEAA/DMF and PNIPA-b-PNEAA/THF solution, suggesting the segregation of the PNIPA-b-PNEAA chains in DMF and THF. The time correlation function of the star polymer/acetonitrile solution can be well fitted with a stretched exponential function (g_2_ = 1 + exp[-2(Γτ)^β^]) with the stretch exponent β = 1, indicating that only a single dynamic is present (where Γ is the decay rate of the time correlation function).

**
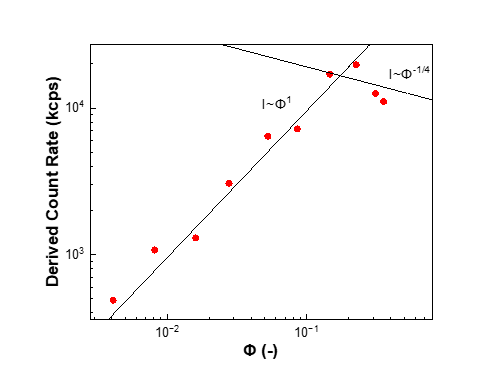
**

Fig. S6 Scattering intensity (*I*) of PNIPA-b-PNEAA-N_3_ in Acetonitrile for various polymer volume fractions ($\Phi=0.004, 0.008, 0.016, 0.028, 0.052, 0.085, 0.146, 0.226, 0.311, and 0.356$). The solid lines represent the theoretical scaling relations for a dilute (I ~ $\Phi$^1^) and semidilute solution (I ~ $\Phi$^−1/4^) in a good solvent, respectively.
